# Development of a dietary formulation of the SHetA2 chemoprevention drug for mice

**DOI:** 10.1007/s10637-017-0550-0

**Published:** 2017-12-22

**Authors:** Doris M. Benbrook, Naveena B. Janakiram, Vishal Chandra, Gopal Pathuri, Venkateshwar Madka, Nicole C. Stratton, Chioniso P. Masamha, Cassadie N. Farnsworth, Lucila Garcia-Contreras, Manolya Kukut Hatipoglu, Stan Lighfoot, Chinthalapally V. Rao

**Affiliations:** 1 Section of Gynecologic Oncology, Department of Obstetrics and Gynecology, College of Medicine, Stephenson Cancer Center, University of Oklahoma Health Sciences Center, 975 NE 10th St., BRC 1217A, Oklahoma City, OK 73104 USA; 2 Center for Cancer Prevention and Drug Development, Stephenson Cancer Center, University of Oklahoma Health Sciences Center, 975 NE 10th St., Oklahoma City, OK 73104 USA; 3 Hematologic Oncology Section, College of Medicine, Center for Cancer Prevention and Drug Development, Stephenson Cancer Center, University of Oklahoma Health Sciences Center, 975 NE 10th St., Oklahoma City, OK 73104 USA; 4 VA Medical Center, Oklahoma City, OK 73104 USA; 5 Present Address: Butler University, 4600 Sunset Avenue, Indianapolis, IN 46208 USA; 6 Cameron University, 2800 W Gore Blvd, Lawton, OK 73505 USA; 7 Department of Pharmaceutical Sciences, College of Pharmacy, Stephenson Cancer Center, University of Oklahoma Health Sciences Center, 1110 N. Stonewall, Oklahoma City, OK 73117 USA

**Keywords:** Dietary formulation, SHetA2, Kolliphor HS15, Intestinal absorption, Toxicity, Cyclin D1

## Abstract

Development of cancer chemoprevention compounds requires enhanced consideration for toxicity and route of administration because the target population is healthy. The small molecule drug, SHetA2 (NSC 726189), exhibited in vivo chemoprevention activity and lack of toxicity when administered by oral gavage. Our objective was to determine if a dietary formulation of SHetA2 could achieve effective tissue drug levels without toxicity. C57bl/6 J mice were monitored on modified American Institute of Nutrition (AIN)76A diet mixed with SHetA2 in a 3:1 ratio with Kolliphor HS15, a self-emulsifying drug delivery system (SEDDS) to deliver 37.5, 62.5, 125, 187 or 250 mg SHetA2/kg/day. Blood and tissues were evaluated after 1, 3 and 6 weeks. The 187 mg/kg/day dose was identified as optimal based on achievement of maximum blood and tissue drug levels in the effective micromolar range without evidence of toxicity. The 250 mg/kg/day group exhibited lower drug levels and the highest intestinal drug content suggesting that an upper limit of intestinal absorption had been surpassed. Only this highest dose resulted in liver and kidney function tests that were outside of the normal range, and significant reduction of cyclin D1 protein in normal cervical tissue. SHetA2 reduced cyclin D1 to greater extents in cancer compared to non-cancer cell cultures. Given this differential effect, optimal chemoprevention without toxicity would be expected to occur at doses that reduced cyclin D1 in neoplastic, but not in normal tissues. These findings support further development of SHetA2 as a chemoprevention agent and potential food additive.

## Introduction

Current therapeutic strategies for gynecologic cancers incorporate various combinations of surgery, radiation, chemotherapy and molecularly-targeted drugs, which commonly cause severe patient morbidity [1, 2]. In contrast, cancer prevention strategies, if successful, could reduce the morbidity and mortality caused by both the cancer and the therapeutic treatments. In cancer prevention schemes, only minimal-to-no toxicity will be acceptable to the target population. While individuals at high risk for development of gynecologic cancer, such as women with BRCA gene mutations or human papillomavirus (HPV) infections, might be willing to accept a small amount of toxicity to reduce their risk of cancer development, the general population will have a higher benefit-to-risk-of-side-effects tolerance. Similarly, patients undergoing secondary cancer prevention schemes after primary therapy are likely to accept a low level of toxicity to reduce cancer recurrence.

The approach of using natural or synthetic compounds to prevent cancer (chemoprevention) has been proven effective in multiple experimental models and clinical trials, however side effects caused by long-term continuous use of chemoprevention agents, such as selective estrogen response modulators (SERMS), have limited their acceptability in healthy individuals [3, 4]. The route of administration of chemoprevention agents is another significant consideration. Direct application to accessible areas of the body or oral administration are preferred over more invasive methods, such as injection, especially in situations where long-term continuous use is warranted. Only an orally-available chemoprevention compound that exhibits no evidence of toxicity in large population-based studies could be considered for use as a food additive.

**Fig. 1:**
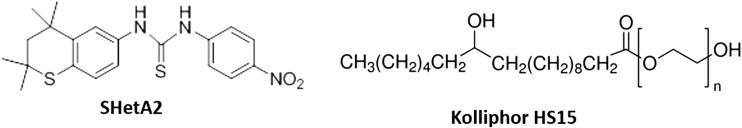
Chemical structures of SHetA2 and Kolliphor HS15

SHetA2 (NSC 726189, 1-(4-nitrophenyl)-3-(2,2,4,4-tetramethylthiochroman-6-*yl*)thiourea, Fig. 1) is a small molecule drug that has met these preferred requirements for a chemoprevention agent in pre-clinical studies. This drug induces G1 cell cycle arrest and apoptosis in cancer cells, while its effects on healthy cells is limited to G1 cell cycle arrest [5–11]. The molecular mechanism of apoptosis involves SHetA2 binding to mortalin (HSPA9) and causing release of p53 [12]. Extensive preclinical studies found that SHetA2 had no evidence of mutagenicity, carcinogenicity or teratogenicity [6, 13, 14]. Detailed analysis of toxicity endpoints during 28-day oral SHetA2 dosing with 0, 100, 500, and 2000 mg/kg/day in rats and 0, 100, 400, and 1500 mg/kg/day in dogs deduced a No Observed Adverse Effect Level (NOAEL) of 500 mg/kg/day in rats and >1500 mg/kg/day in 28-day dogs [15]. These doses are 17 to 50 fold above the 30 mg/kg/day oral dose of SHetA2 shown to reduce the incidences and sizes of colon and small intestinal tumors in both genders in the APC^min/+^ mouse model [16]. This large therapeutic window suggests that SHetA2 could be developed as a chemopreventive agent with an acceptable benefit-to-risk ratio for use in the general population. The major metabolites of SHetA2 were found to have been hydroxylated or glutathionylated, however hydroxylated analogs are inactive and no evidence of glutathione depletion or liver toxicity was observed in the 28-day toxicity testing [15, 17]. Bioavailability of SHetA2 is low, however suspension of SHetA2 in Kolliphor HS15 (Fig. 1), a self-emulsifying drug delivery system (SEDDS), was shown to increase the bioavailability by 10 -fold over suspension in 1% aqueous methylcellulose/0.2% Tween 80 [15]. The objective of this study was determine if various doses of SHetA2 could be administered in a dietary formulation containing Kolliphor HS15 to achieve chemoprevention-effective serum and tissue drug levels without toxicity.

## Materials and methods

### Materials

Dietary ingredients based on the American Institute of Nutrition (AIN) diet included casein, corn starch, dextrose, corn oil, alphacel/cellulose, DL-methionine, mineral mix, vitamin mix and choline bitartrate were purchased from Bio-Serv (Flemington, NJ, USA). Kolliphor HS 15 was purchased from Sigma-Aldrich (St. Louis, MO, USA). SHetA2 compound was kindly provided by the United States National Cancer Institute (NCI) Rapid Access to Intervention Development (RAPID) program. The human ovarian cancer SKOV-3 cell line was purchased from American Type Culture Collection (Manassas, VA) and the human ovarian cancer cell line A2780 and the immortalized ovarian surface epithelial cells (IOSE-80) were gifts from Michael Birrer, MD, PhD (Harvard Medical School, Boston, MS, USA). Tissue culture media reagents (RPMI 1640 Medium 199, MCDB105, L-glutamine, sodium pyruvate, HEPES buffer), a protease inhibitor cocktail and dimethylsulfoxide (DMSO) were purchase from MilliporeSigma (St. Louis, MO, USA). Fetal Bovine Serum (FBS) was purchased from Serum Source International (Charlotte, NC, USA). A Cyclin D1 ELISA kit was purchased from Cloud-Clone Corp. (Houston, TX, USA). The 0.2 μm filters were purchased from Agilent (Santa Clara, CA, USA). The m-PER and t-PER protein extraction reagents, bicinchoninic acid (BCA) protein concentration measurement kit and an antibiotic/antimycotic mixture were purchased from Thermo-Fisher (Waltham, MA, USA). the Polyvinylidene difluoride (PVDF) membrane, the Transfer Blot Turbo Transfer Pack containing the PVDF membrane, and the Clarity Western ECL Substrate Chemiluminescence detection system were purchased from Bio-Rad (Hercules, CA, USA). An antibody specific for cyclin D1 was purchased from BD Pharmingen (San Jose, CA, USA). Luminol reagent and antibodies specific for GAPDH and β-actin and secondary antibodies conjugated to horse-radish peroxide (HRP) were purchased from Santa Cruz (Dallas, TX, USA). Luminol reagent was purchased from GE Healthcare Life Sciences (Pittsburgh, PA, USA).

### Diet formulation

A semi-purified diet based on modified AIN-76A (Casein, 20%; Corn Starch, 52%; Dextrose, 13%; Corn oil, 5.0%; Alphacel/cellulose, 5.0%; DL-Methionine, 0.3%; Mineral mix AIN, 3.5%; Vitamin mix, AIN, 1.0%; and Choline bitartrate, 0.2%) was prepared in the University of Oklahoma Health Sciences Center (OUHSC) Rodent Barrier Facility. The ingredients were mixed thoroughly to assure that all micronutrients were uniformly distributed in the diet. In order to assure that SHetA2 was uniformly distributed in the diet, the agent was pre-mixed with a small quantity of control diet in a food mixer, added to a pre-weighed amount of control diet in a Hobart Mixer and mixed thoroughly for about 45 min. Both control and experimental diets were prepared weekly and stored at 4 °C in the cold room.

### Animal, dosing, observation and necropsy

**Fig. 2:**
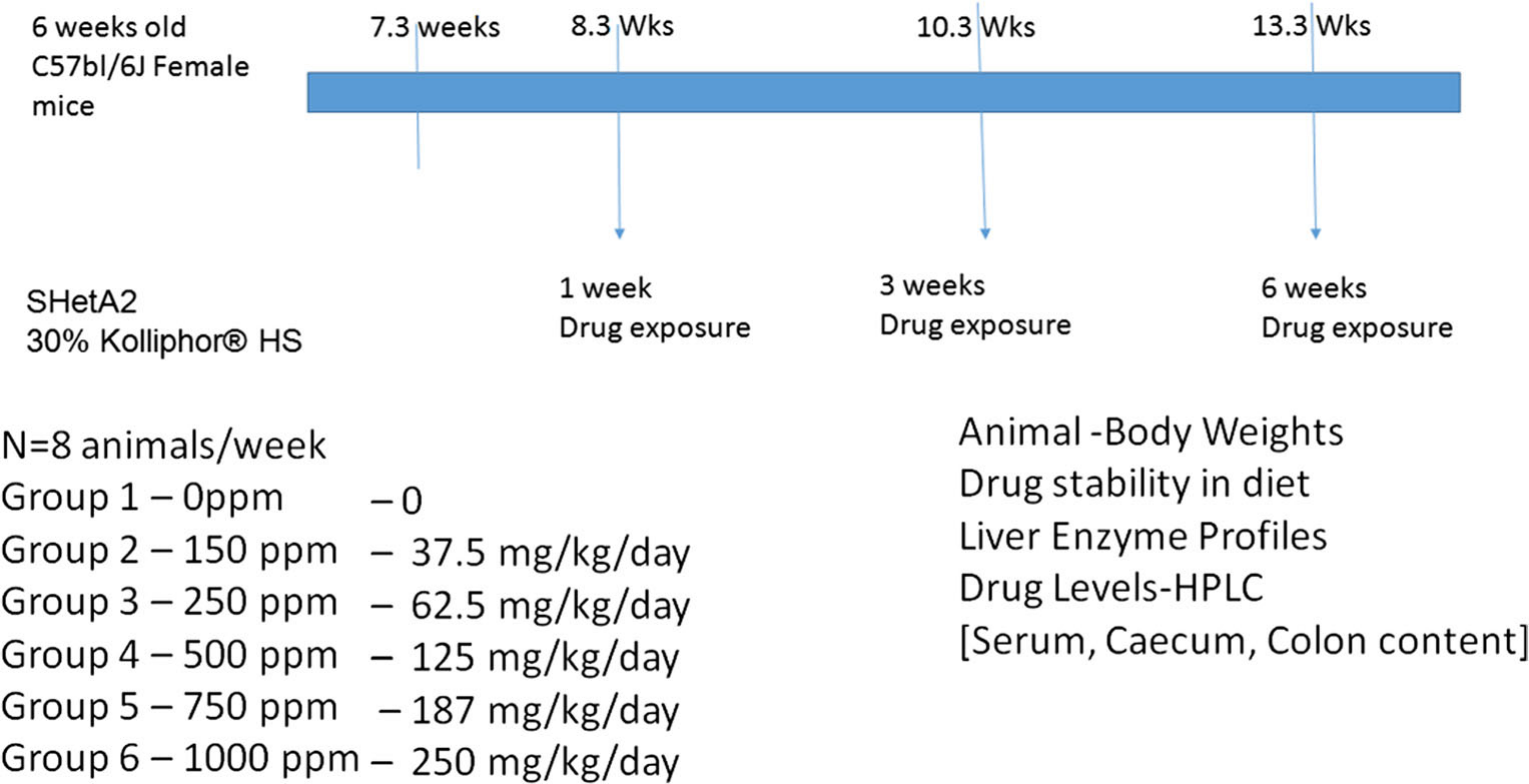
Experimental design

The experimental design is illustrated in Fig. 2. C57BL/6 J female mice were bred at OUHSC Rodent Barrier Facility. The research was performed under the animal protocols approved by the OUHSC Institutional Animal Care and Use Committee (IACUC) and in compliance with the National Institutes of Health guide for care and use of Laboratory animals (NIH Publication No. 8023, revised 1978). Experimental animals were housed in ventilated cages under standardized conditions (21 °C, 60% humidity, 12-h light/12-h dark cycle, 20 air changes/h) in the OUHSC rodent barrier facility. Mice were allowed ad libitum access to the respective diets and to automated tap water purified by reverse osmosis. At 6–7 weeks of age, groups of 24 mice were fed control diet or the experimental diets containing 150 ppm, 300 ppm, 500 ppm, 750 ppm, and 1000 ppm SHetA2 for six weeks. The diet was replenished with freshly-made diet twice per week. All animals were weighed once weekly until termination of the study. Organs were collected and weighed at necropsy. Eight animals from each group were euthanized 1, 3 and 6 weeks after initiating the diet administration and necropsied to collect blood, livers, kidneys, intestines, ovaries, fallopian tubes, uterine horns, and cervices. The blood was collected in microfuge tubes and immediately processed into serum using standard protocols. Due to the low volume of serum that can be collected from and individual mouse, each mouse produced a single serum specimen that was used either for one of the individual toxicity endpoints or for the drug level measurement. The tissues and serum were placed in labeled cryovials, snap frozen in liquid nitrogen and stored at -80 °C until further analysis.

### Serum specimen preparation for HPLC analysis

The zero control (internal standard, IS only) was prepared by adding a 10 μL aliquot of 5 μg/mL 2, 3-diphenylquinoxaline (internal standard, IS) to a 1.5 ml amber colored Eppendorf tube, and then adding an additional 90 μL of serum. The tube was vortexed for 30 s, 80 μL of chilled acetonitrile was added and then the tube again was vortexed vigorously on multi-shaker for 10 min followed by centrifugation at 16,100 xg for 10 min at 4 °C. The supernatant was filtered through 0.2 μm filter and then injected (70 μL) into an HPLC.

### Cecum and colon content specimen preparation for HPLC analysis

The frozen tissue (cecum) and colon content were rapidly thawed. The tissue was opened with a razor blade or scissors and approximately 50 mg of the content was transferred into 2 ml brown microcentrifuge tubes (VWR, Radnor, PA, USA), and then submerged in an ice bath. One mL of cold acetonitrile was added and vortexed for 10 min on analog multi-tube vortexer (VWR, Radnor, PA, USA). The homogenate was centrifuged at 16000 rcf at 4 °C for 5 min. The supernatant was filtered through Captiva filtration (0.2 μm filter. Agilent, Santa Clara, CA, USA). 5 or 10 μl of filtrate was injected into HPLC. The entire process was carried out under incandescent lights and under 4^ o^C.

### HPLC analysis of SHetA2 in serum and in cecum and colon content

Serum, cecum content and colon content reversed-phase HPLC analyses were performed on a Shimadzu HPLC equipped with a LC-10ADvp pump, SCL 10Avp system controller and a SPD-M10Avp PDA detector. All the operations were controlled by Shimadzu EZ start 7.2.1 software in Windows XP system. The PDA detector was set between 190 to 800 nm and analysis was done at 341 nm. HPLC solvents consisted of 65% Acetonitrile and 35% Distilled water. An isocratic method with flow rate 0.18 ml/min used with total run time 40 min (serum samples), and 20 min (cecum and colon content samples). The injection volume was 70 μL for serum samples and 5 or 10 μl for cecum content and colon content samples. A XBridgeTM C18 (Waters, BEH 130, 3.5 μm, 2.1 × 150 mm) column with a Precolumn XBridge (BEH C18 Sentry Guard Cartridge, 130 Å, 3.5 μm, 2.1 mm × 10 mm) was used. Retention times for DQ (IS) is 9.6 min, and SHetA2 is 11.7 min.

### HPLC analysis of SHetA2 in tissue

HPLC analysis was performed using a Waters Alliance HPLC System with a V_ydac_ 201 TP C_18_ 5 μ (250 mm × 2.1 mm) column equipped with a guard column (V_ydac_ 201 TP, Grace), and a UV detector set at 341 nm. The mobile phase consisted of acetonitrile:water (80:20, *v*/v).The flow rate was 0.3 mL/min with a retention time 3.65 min and the total run time of each analysis was 7 min at ambient temperature. The injection volume was 10 μL. Empower software was used for the integration of the peak areas.

### Evaluation of general toxicity

Animals were examined daily for any external signs of toxicity that would be predicted to shorten the natural life span of the animal, such as roughened coat, chromodacryorrhea, rhinitis, and prostration. Body weights were recorded at weekly intervals. Organ-to-body weight ratios were determined at weeks 1, 2 and 3. Sections of paraffin-embedded organ specimens were stained with hematoxylin and eosin (H&E) for pathology review. Three serum specimens from each of the groups treated for 6 weeks with 500 ppm, 750 ppm and 1000 ppm SHetA2 diets were evaluated for liver and kidney function tests: alanine amino transferase (ALT), aspartate amino transferase (AST), alkaline phosphatase (ALKP) and blood urea nitrogen (BUN) and creatinine (CRE) using an IDEXX Catalyst Dx Chemistry Analyzer.

### Tissue specimen preparation

For SHetA2 extraction, tissues were rapidly thawed and washed thoroughly with sterile saline solution. Tissues were then weighed and homogenized in an acetonitrile:saline mixture (80:20, v/v) using a bead homogenizer. Samples of 2 ml/g of tissue were centrifuged at 14000 rpm for 10 min. Supernatants were collected and loaded onto a Captiva filtration plate and filtered by vacuum. Filtrates were collected and injected directly into the HPLC for analysis of drug levels.

For Western blot and Enzyme linked immunosorbent assay (ELISA) analysis, necropsy cervical tissues were homogenized in ice-cold t-PER protein extraction solution containing a protease inhibitor cocktail and phosphatase inhibitor cocktail using a homogenizer. The homogenates were incubated on ice for 1 h with occasional shaking and then centrifuged at 13,000×g for 15 min at 4 °C. Protein concentrations in supernatants were determined using the BCA assay according manufacturer’s instructions.

### Maintenance and treatment of tissue cultures

Ovarian cancer cell lines (SKOV3 and A2780) were maintained in RPMI 1640 with L-glutamine (supplemented with 1 mmol/L sodium pyruvate, 1 mmol/L HEPES buffer, antibiotic/antimycotic and 10% FBS) and IOSE-80 cultures were maintained Medium 199/ MCDB105 (1:1) supplemented with antibiotic/antimycotic and 15% FBS. Cells were treated with 10 μM SHetA2 dissolved in DMSO or the same volume of DMSO solvent for 24 h prior to extracting proteins from cells with the m-PER reagent. Protein concentrations in the extracts were quantified with the BCA kit.

### Western blot analysis of cyclin D1 protein levels

Equal amounts of proteins from extracts of animal specimens or cell cultures experiments were electrophoresed through a 10% SDS-PAGE gel and transferred to a PVDF membrane for the cell culture extracts. A 12% SDS-PAGE gel and the Turbo-Blot Turbo Transfer Pack was used for the animal tissue extracts. The membranes were blocked for one hour in 5% skim milk and then incubated overnight at 4 °C with the cyclin D1 primary antibody at 1:1000 dilution. The next day, the membranes were rinsed and incubated with the HRP-conjugated secondary antibody for 1 h. The specific signal was detected with Luminol reagent and exposure to X-ray negatives for the membranes containing the cell culture extracts and with Clarity Western ECL Substrate and BioRad ChemiDoc Touch Imaging System for the membranes containing the animal tissue extracts. To confirm equal loading, the membranes were stripped and re-probed the GAPDH antibody at 1:1000 dilution for 2 h at room temperature for cell culture extracts or the β-actin antibody at 1:2000 overnight at 4 °C for the animal tissue extracts.

### ELISA of cyclin D1 protein levels in tissues

Total protein was isolated from tissues in ice-cold m-PER protein extraction solution containing protease inhibitor cocktail and phosphatase inhibitor cocktail by using a homogenizer. Equal amounts of protein (5 μg) were added to an ELISA plate by and evaluated following manufacturer’s instructions. The ODs were measured with a Synergy H1 ELISA plate reader at 450 nm. The experiments were performed in duplicate.

### Statistical analysis

Determination of normality and statistical analysis was performed with GraphPad Prism 6. The *p* values of less than 0.05 were considered statistically significant.

## Results

### Stability and consumption of SHetA2 in the dietary formulation

**Fig. 3:**
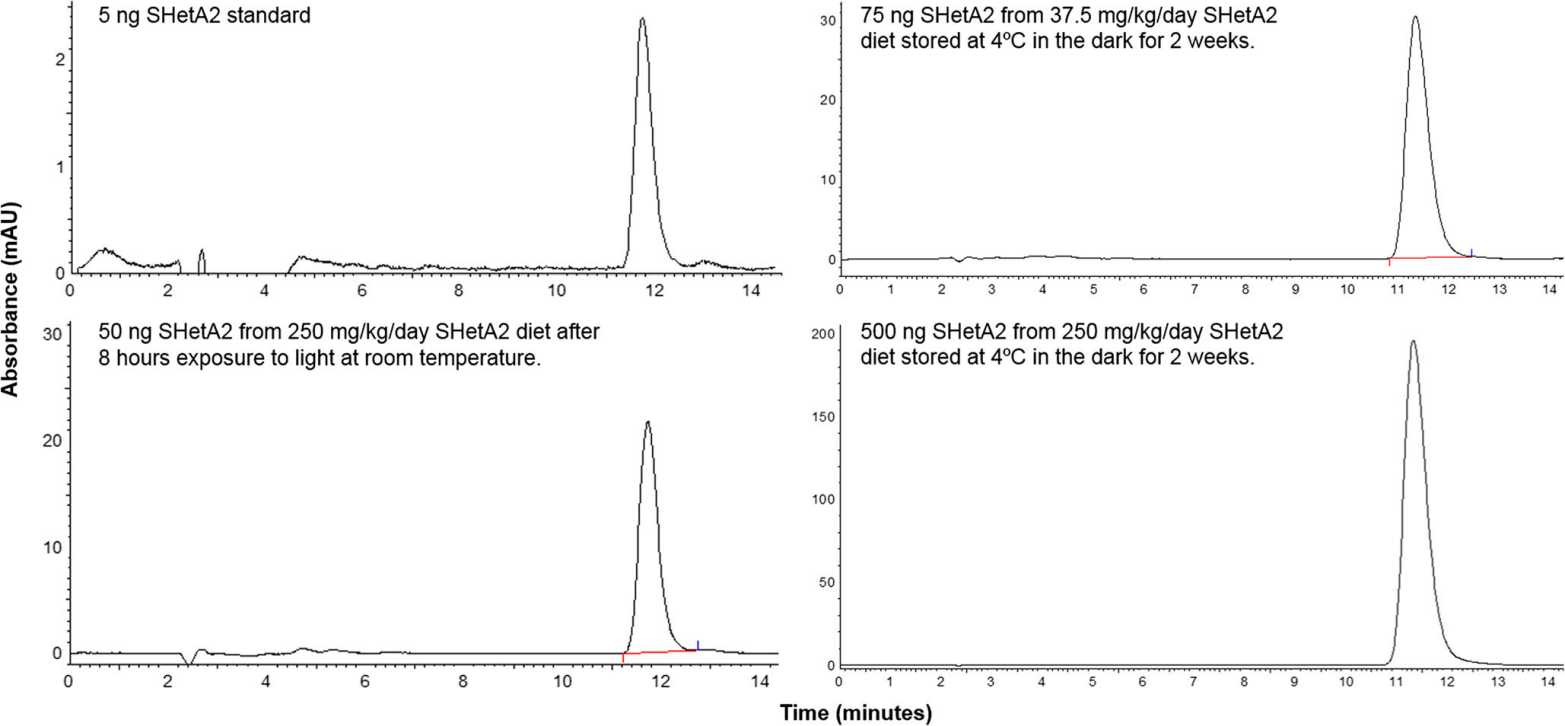
HPLC/UV Analysis of SHetA2 Stability in diets under various conditions. HPLC chromatograms of SHetA2 Standard and SHetA2 extracted from SHetA2-containing diets exposed to various light and temperature conditions as indicated

SHetA2 in the dietary formulation was found to be stable after exposure to light at room temperature for 8 h, or stored at 4 °C in the dark for two weeks, with no variation from the initial starting material or presence of peaks corresponding to drug degradation products observed (Fig. 3). Throughout the experiment, the diet was prepared twice per week and stored at 4 °C in the dark to assure drug stability.

### Evaluation of general toxicity

**Fig. 4:**
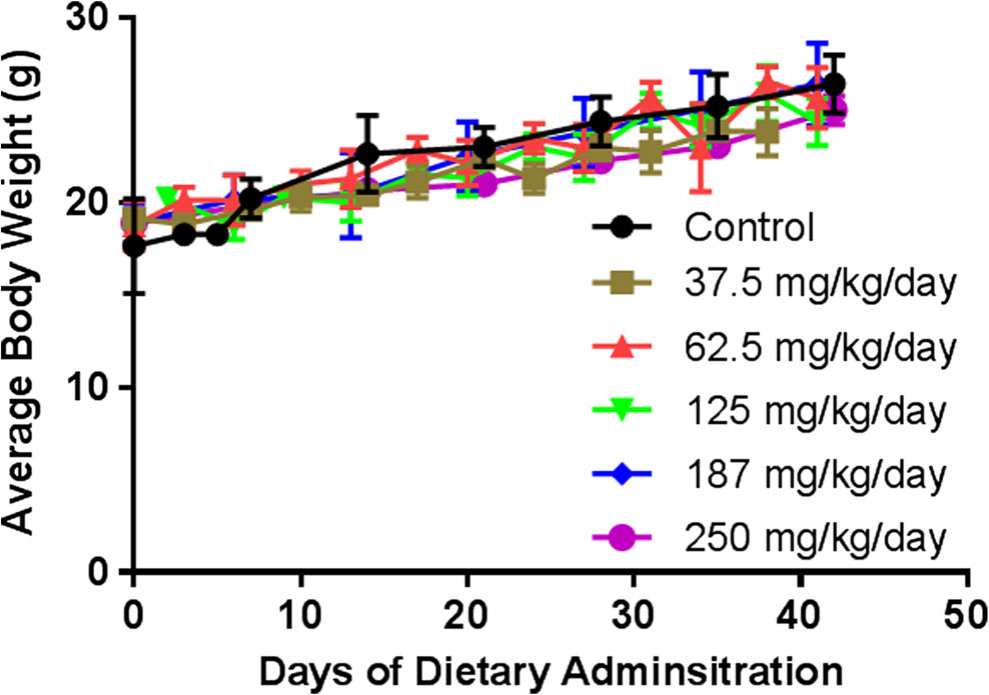
Animal body weights during treatment period. Individual body weights were measured weekly. The averages and standard error of the mean for each dose group are plotted versus treatment time

Based on an estimated average of 5 g of diet consumed per day, the animals assigned to the 150, 250, 500, 750 or 1000 ppm dietary SHetA2 groups consumed 37.5, 62.4, 125, 187 or and 250 mg/kg/day, respectively, over the 1, 3 and 6 week treatment periods. No significant differences were observed in growth rates between the various treatment groups (Fig. 4, Linear regression comparison of slopes, F = 0.944, *P* = 0.453) or between the organ-to-body weight ratios between the various groups (Table 1. Repeated Measures ANOVA with Dunnett correction for multiple comparisons, Liver: F = 0.59, *p* = 0.55; Kidney: F = 3.27, *P* = 0.16; Spleen: F = 1.35, *P* = 0.37).

**Table 1:** Average and standard errors of organ-to-body weight ratios

	Treatment groups (mg/kg/day)					
	Control	37.5	62.5	125	187	250
Week 1						
Liver	45.89 ± 2.72	43.85 ± 2.60	40.17 ± 0.65	46.73 ± 2.35	40.91 ± 1.72	41.23 ± 1.34
Kidney	13.03 ± 0.03	13.39 ± 0.39	11.67 ± 0.55	12.59 ± 0.53	11.57 ± 0.21	11.45 ± 0.48
Spleen	3.43 ± 0.15	4.22 ± 0.37	3.25 ± 0.21	3.64 ± 0.28	3.70 ± 0.21	3.50 ± 0.44
Week 3						
Liver	37.49 ± 4.35	45.21 ± 2.78	41.22 ± 2.47	44.27 ± 1.49	40.53 ± 2.41	38.97 ± 0.53
Kidney	13.40 ± 1.43	12.43 ± 0.33	12.94 ± 0.68	11.30 ± 0.41	10.98 ± 0.38	10.57 ± 0.33
Spleen	4.14 ± 0.29	4.47 ± 0.50	3.03 ± 0.15	3.22 ± 0.17	3.26 ± 0.22	3.18 ± 0.20
Week 6						
Liver	48.42 ± 4.58	35.96 ± 2.79	40.22 ± 1.91	39.87 ± 2.44	41.14 ± 3.42	43.22 ± 1.68
Kidney	11.79 ± 0.71	11.32 ± 0.39	10.94 ± 0.62	11.07 ± 0.67	11.44 ± 0.44	10.78 ± 0.47
Spleen	2.74 ± 0.77	3.25 ± 0.25	3.00 ± 0.47	3.54 ± 0.49	3.23 ± 0.24	3.84 ± 0.35

Liver and kidney function tests were performed on blood specimens of the three highest dose groups collected after 6 weeks of dietary administration (Table 2). BUN levels were slightly higher than normal for the 125 mg/kg/day group and slightly lower than normal in the 250 mg/kg/day group. Ast and Alkp levels were reduced in the 250 mg/kg/day group.

**Table 2:** Kidney and liver function tests

Endpoint	Normal	Control	125 mg/kg/day	187 mg/kg/day	250 mg/kg/day	ANOVA
Bun (mg/dl)	18–29	28 ± 2	**33 ± 1**	23 ± 1	**16 ± 0**	F = 1.4, *p* = 0.31
Cre (mg/dl)	0.2–0.8	< 0.2	< 0.2	< 0.2	< 0.2	Not applicable
Alt (U/L)	28–132	45 ± 3	101 ± 35	34 ± 5	108 ± 68	F = 1.6, *p* = 0.27
Ast (U/L)	59–247	151 ± 48	172 ± 23	90 ± 8	**9 ± 5**	F = 7.3, *p* = 0.01
Alkp (U/L)	62–209	77 ± 21.7	109 ± 12.1	115 ± 18	**< 20**	Not applicable

Abnormal levels are bolded

**Fig. 5:**
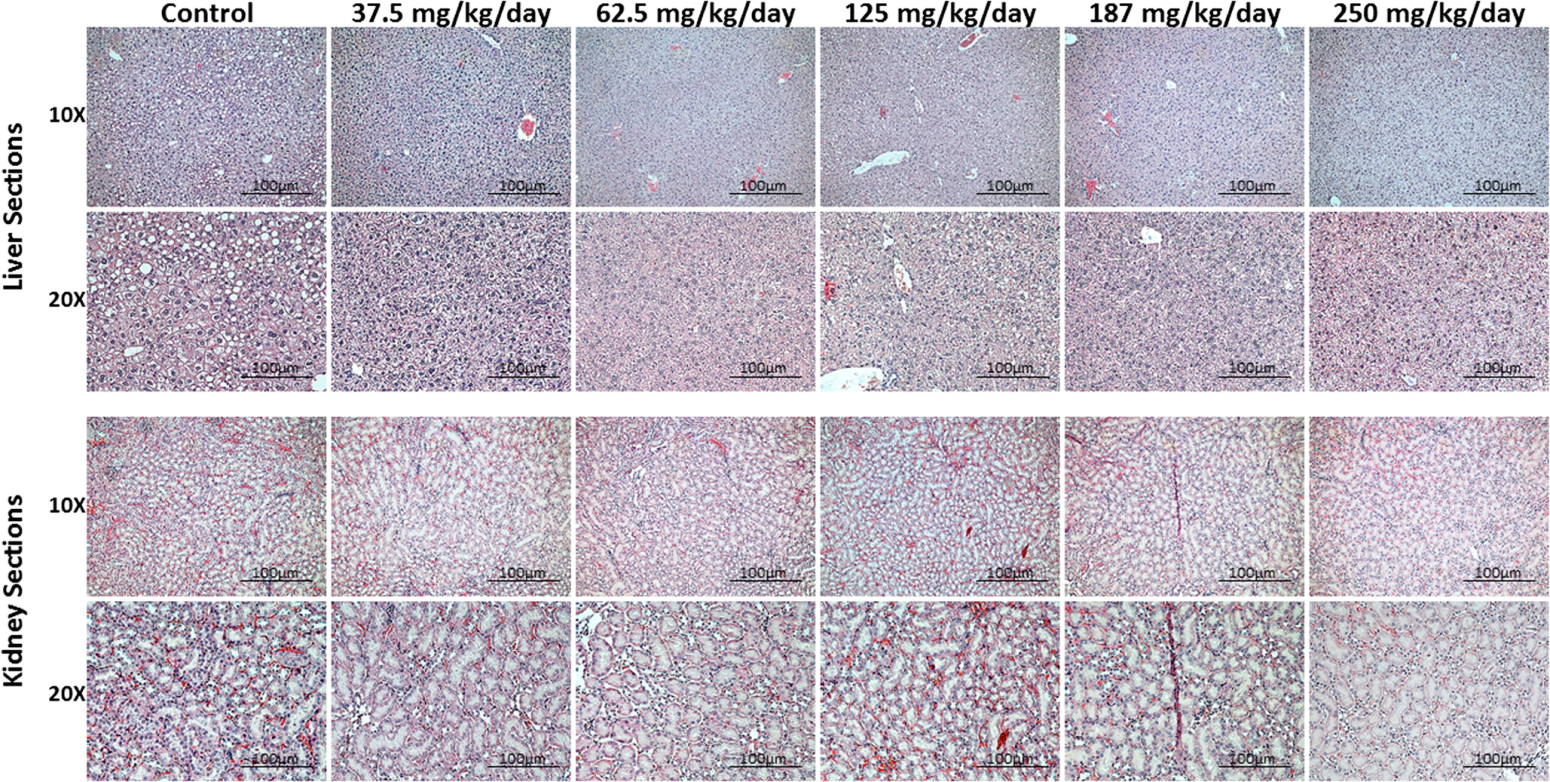
Histology of livers and kidneys across all dose groups. Representative images of H&E stained sections of liver and kidney tissues from each dose group are shown

An experienced pathologist (S.L.) evaluated H&E stained sections from each of the kidney specimens in blinded manner and reported that all specimens appeared normal. Histologies of the kidney and liver specimens from each dose group are shown in Fig. 5. The proximal and distal tubules, glomeruli, arterioles and venules and glomeruli of the kidney were normal. The kidney glomeruli were well-formed with no inflammation, peri-glomerular fluid or hyalinization. The livers appeared normal with no signs of apoptosis, necrosis or inflammation. The control group exhibited some liver steatosis (accumulation of fat) at a level of 2+. An occasional fat cell was present in livers from the treatment groups, however this was within normal limits and none of the treatment group livers from exhibited 2+ steatosis.

### Evaluation of serum and tissue drug levels

**Fig. 6:**
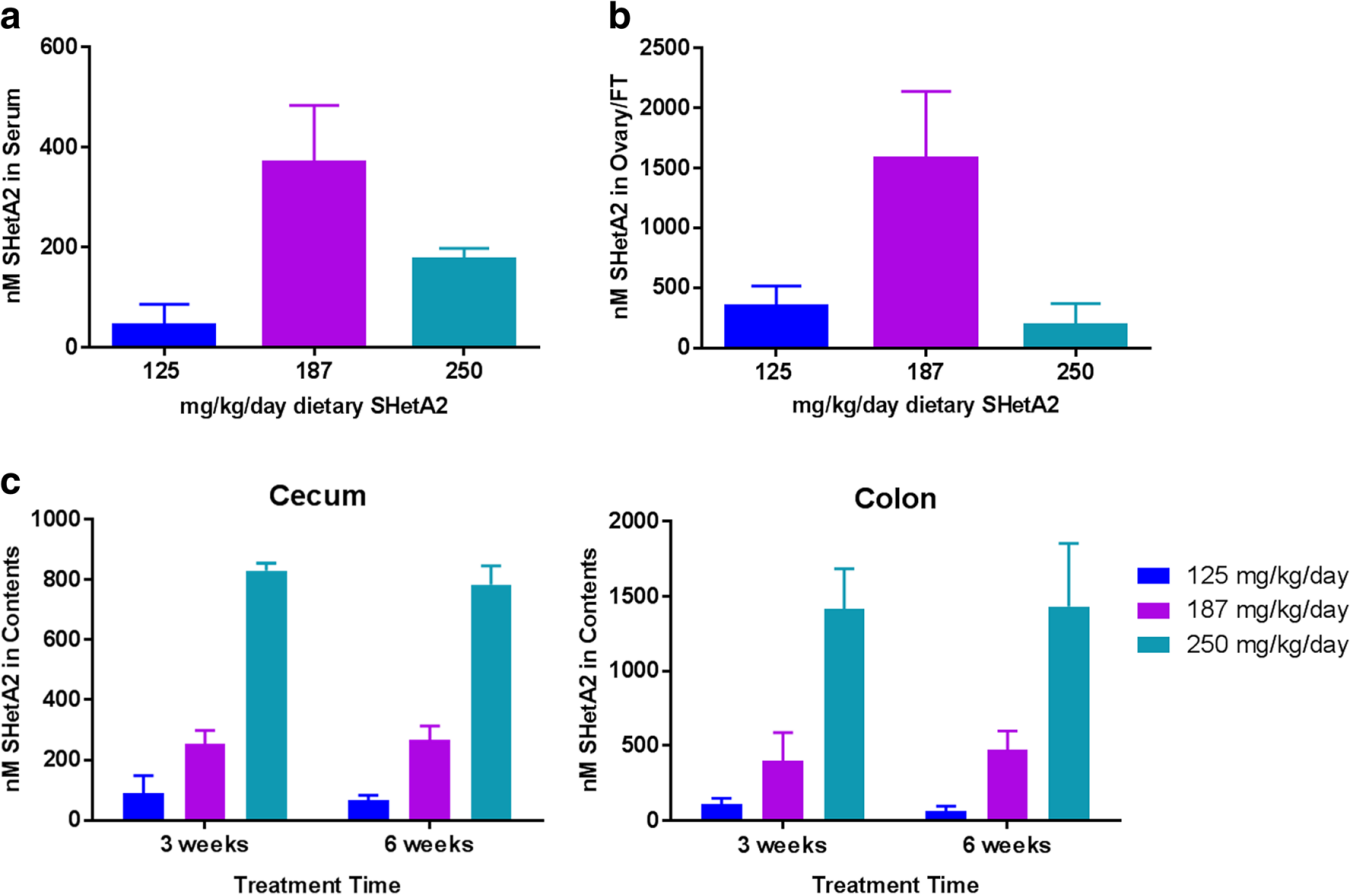
SHetA2 drug levels in treated animals. HPLC/UV was used to measure SHetA2 extracted from serum (**a**), ovarian and fallopian tube combined tissues (Ovary/FT) (**b**) and the contents of cecum and colon (**c**). Bars represent the average and standard error of the mean of biological replicates A revised figure has been emailes with Ceacum changed to Cecum and the 150 changed to 250 (make correction to legend 250 mg/kg/day -not 150 mg/kg/day)

Gynecologic tissues were chosen for evaluation in this study, because SHetA2 is being developed currently for prevention of ovarian, cervical and endometrial cancers. After 3 weeks of treatment, SHetA2 levels were undetectable in the serum and combined ovarian and fallopian tube (Ovary/FT) specimens from mice that received the 37.5 and 62.5 mg/kg/day doses and measurable in the 125, 187 and 250 mg/kg/day dose groups (Fig. 6a, b, respectively). The highest serum drug levels were observed in the 187 mg/kg/day dose group, which were significantly higher compared to the drug levels measured in the 125 mg/kg/day dose group (One-way ANOVA, *p* = 0.032; and Tukeys multiple comparisons *p* value between 125 and 187 mg/kg/day, p value <0.05). The 250 mg/kg/day dose group exhibited lower drug levels compared to the 187 mg/kg/day dose group, but the difference was not statistically significant (One-way ANOVA with Tukey’s multiple comparison p value >0.05). The drug levels in the Ovary/FT specimens exhibited the same pattern with the highest concentrations in the 187 mg/kg/day group, however there were insufficient numbers of specimens available for statistical analysis and the tissue levels were much higher than the blood levels. The drug significantly accumulated in the cecum and colon contents in a dose-dependent manner, however there were no significant differences in the levels measured between 3 and 6 weeks of treatment (Fig. 6c). A two-way ANOVA demonstrated significant differences between doses (*p* = 0.001 for caecum contents and *p* = 0.016 for colon contents), but not between the treatment times (*p* = 0.066 for the caecum contents and *p* = 0.839 for colon contents).

### Evaluation of gynecologic tissue toxicity

**Fig. 7:**
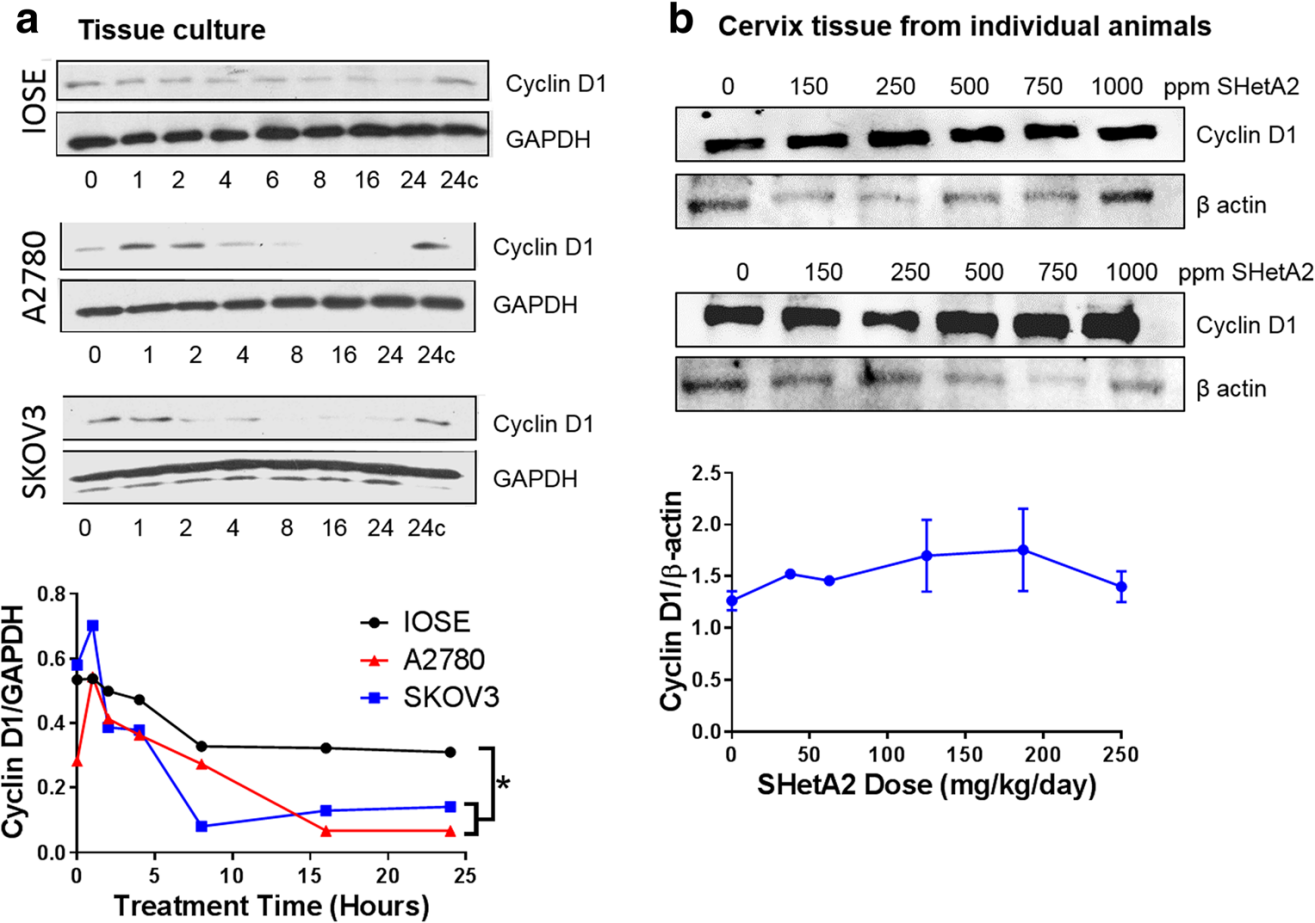
Western blots of SHetA2 effects on cyclin D1 levels. Western blots of proteins extracted from IOSE cultures and the human ovarian cancer A2780 and SKOV3 cell lines treated with 10 μM SHetA2 for the indicated times or with the same volume of DMSO vehicle for 24 h (24c) were probed with cyclin D1 or GAPDH (**a**). Western blots of proteins extracted from cervical tissues of mice given the indicated dietary SHetA2 doses for 6 weeks were probed with cyclin D1 and GAPDH (**b**). Densitometric scans were used to determine the band intensities and the cyclin D1 intensity was normalized to the GAPDH intensity (Cyclin D1/GAPDH) and plotted against the treatment time or dietary dose. The asterisk indicates significant differences in cyclin D1 reduction in the ovarian cancer cell lines compared to the IOSE cells (Repeated Measures ANOVA with Dunnett correction for multiple comparisons)

SHetA2 has been shown to induce G1 cell cycle arrest in normal and cancer cells, and the mechanism has been shown to involve phosphorylation, ubiquitination and proteolytic degradation of cyclin D1 [10]. Use of cyclin D1 as a pharmacodynamic (PD) endpoint biomarker of SHetA2 chemoprevention activity was supported by the observation of a SHetA2 dose-dependent decrease of polyp cyclin D1 levels in association with reduction of the number and sizes of polyps in the APC^min/+^ model of colorectal cancer [16]. The effects of SHetA2 on molecular endpoints in cancer cells are much greater than its effects in healthy cells, which translates into the lack of observed toxicity [6, 7, 15, 18], however differential effects on cyclin D1 specifically have not yet been evaluated. In this study, Western blot analysis demonstrated a dose-dependent reduction of cyclin D1 in human ovarian cancer cell lines, but not in non-cancerous IOSE cell cultures or in cervical tissues from individual animals treated for 6 weeks with the diet containing various SHetA2 doses (Fig. 7). A repeated measures ANOVA with Dunnett correction for multiple comparisons indicated that the cancer cell lines exhibited significantly greater cyclin D1 reduction compared to IOSE (*p* < 0.05).

**Fig. 8:**
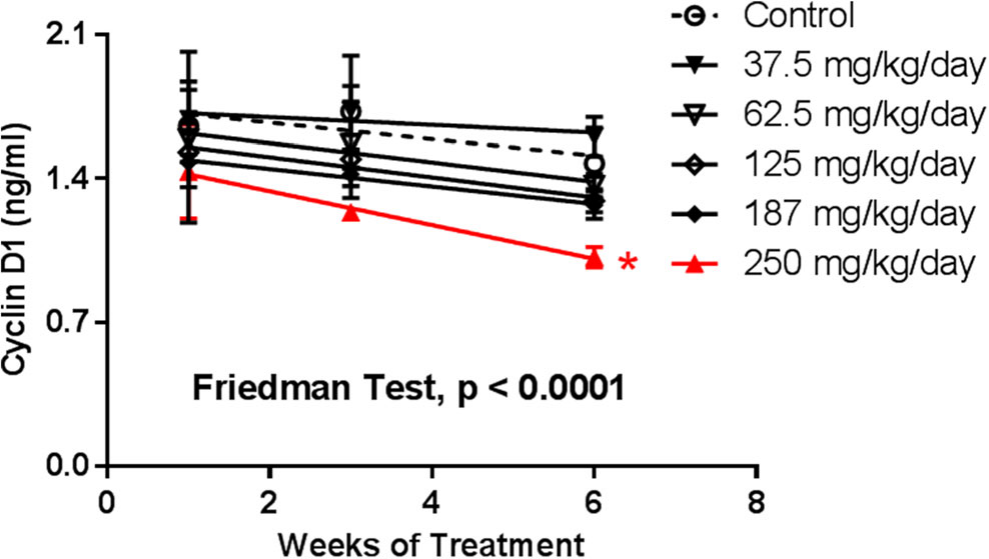
ELISA of cyclin D1 levels in cervical tissues of treated animals. Cyclin D1 in cervical tissue collected at 1, 3 and 6 weeks of SHetA2 dietary administration at the indicated doses are represented as the mean and standard error of 3 biological replicates evaluated by ELISA in duplicate. Linear regression analysis indicated that the slope of the line for cervix was significantly different from zero (*p* = 0.046). The Friedman Test indicated a significant difference between the groups and the posthoc Dunn’s multiple comparison test of all doses compared to the 0 dose indicated that the 1000 ppm dose was significantly lower than the control dose (*P* < 0.05). All graphs were created using GraphPad Prism 6 Software

To increase the sensitivity of cyclin D1 detection, protein extracts of cervical tissues (*N* = 3 per group) collected throughout the study were evaluated by ELISA (Fig. 8). With this increased sensitivity, significant reduction of cyclin D1 was observed only at the highest 250 mg/kg/day dose and the longest treatment time of 6 weeks. The sizes of the ovary/FT specimens were insufficient for Western blot or ELISA analysis of cyclin D1.

## Discussion

This study demonstrated that an AIN76A diet formulation of SHetA2 can achieve nanomolar drug levels in the blood and micromolar drug levels in the ovary/FT tissues. Maximum serum levels of SHetA2 were observed in the 187 mg/kg/day dose group, which was significantly higher than the levels measured in the 125 mg/kg/day group. Reduced levels of serum SHetA2 in the 250 mg/kg/day compared to the 187 mg/kg/day groups is consistent with our previous observations of higher doses having reduced absorption. Pharmacokinetic studies of SHetA2 in mice, rats and dogs indicate that an absorption saturation of oral SHetA2 occurs at about 100 mg/kg body weight/day [15, 17, 19]. The significantly higher levels of SHetA2 observed in the cecum and colon contents of the 250 mg/kg/day group compared to the 187 mg/kg/day group in this study indicate that maximal drug absorption in the lower digestive tract had been achieved below the highest dose tested. An absorption saturation may also explain why oral gavage of SHetA2 in the APC^min/+^ model caused slightly, but not significantly, greater reduction of polyp incidence and size at the lower (30 mg/kg/day) compared to the higher (60 mg/kg/day) [16]. Blood and tissue drug levels had not been measured in that study however.

SHetA2 levels in ovary/FT tissue in this study exhibited the same pattern as the plasma specimens with a maximum level measured in the 187 mg/kg/day dose group. The higher levels of SHetA2 measured in tissues compared to the plasma are consistent with the high hydrophobicity of this molecule. Achievement of micromolar SHetA2 levels in the tissue achieves the concentrations of this drug shown to induce apoptosis in cancer cells, without harming health cells in cell culture studies [6, 7, 18, 20]. Thus, the 187 mg/kg/day SHetA2 diet may be able to exert in vivo anti-cancer effects by causing differential apoptosis in cancer cells.

The 187 mg/kg/day and lower doses appear to be safe based on equivalent total body and organ-to-body weights, and normal organ histology. A greater than normal average BUN level was observed in the 125 mg/kg/day dose group suggestive of dehydration, however a lower than normal average BUN level was observed in the 250 mg/kg/day group suggestive of over-hydration. These abnormal values do not raise serious concerns for kidney toxicity however, because they are not associated with alterations in kidney-to-body weight and the kidney histology appeared normal for all doses. Although the highest SHetA2 dose of 250 mg/kg/day in the diet did not cause loss of body weight or reduced growth, it did cause reduced levels of Ast and Alkp. These alterations are not indicative of liver damage however, because elevated, and not reduced, levels of these enzymes are used as biomarkers of liver toxicity.

Cyclin D1 was chosen as a PD endpoint in this study because its reduction by SHetA2 has been shown to be involved in the mechanism of SHetA2-induced G1 cell cycle arrest [6, 10]. In this study, Western blot analysis was not sufficiently sensitive to detect cyclin D1 reduction by SHetA2 in cervical tissue, however a more-sensitive ELISA method demonstrated significant cyclin D1 reduction in the 250 mg/kg/day dose group and 6 week time point only. Lack of cyclin D1 effects on normal tissue is consistent with our goal of having SHetA2 causing minimal-to-no toxicity. Tissue culture experiments demonstrated that the effect of SHetA2 on cyclin D1 in non-cancerous cells was much reduced in comparison to cancer cell lines. Thus, cyclin D1 reduction in non-cancerous tissue as a surrogate for cancerous tissue is likely to provide an underestimate of the anti-cancer drug effect, and may provide an estimate of potential toxicity. Furthermore, the differential effect of SHetA2 on cancer over non-cancer tissues supports the potential of dietary doses below 250 mg/kg/day (1000 ppm) altering cyclin D1 in pre-cancerous and cancerous tissues, even though they did not reduce cyclin D1 protein in normal cervical tissue. We are currently testing this in the K14-HPV16 genetic mouse model of cervical cancer development.

In conclusion, this study demonstrated that an AIN76A diet formulation of 187 mg/kg/day (750 ppm) SHetA2 mixed with Kolliphor HS15 given ad libitum over 6 weeks can be used to achieve effective tissue drug levels without toxicity in mice. A higher dose accumulated in the lower digestive tract in association with reduced systemic absorption. Cyclin D1 may serve as a pharmacodynamic biomarker for drug action or toxicity based on its differential regulation by SHetA2 in cancer over non-cancer cells. These results support the further study and development of SHetA2 as a food additive for cancer prevention.
